# The relationship between carotenoids and diabetic nephropathy: insights from NHANES

**DOI:** 10.3389/fnut.2025.1584692

**Published:** 2025-07-24

**Authors:** Lei Wang, Tianpu Feng, Ye Tan, Jue Zhang, Ting Zhang, Juan Huo, XiaoXue Song, Xi Lin, Man Li, Wenxue Liang, Qun Ding

**Affiliations:** ^1^Department of Clinical Laboratory, The Affiliated Lianyungang Hospital of Xuzhou Medical University (The First People’s Hospital of Lianyungang), Lianyungang, China; ^2^School of Basic Medical Sciences, Kangda College of Nanjing Medical University, Lianyungang, China; ^3^Department of Endocrinology, The Second People’s Hospital of Lianyungang, Lianyungang, China

**Keywords:** carotenoids, diabetic nephropathy, NHANES, dietary intake, oxidative stress

## Abstract

**Objective:**

Diabetic nephropathy (DN) poses significant health risks and imposes a substantial global disease burden. The association between carotenoid intake and DN remains unclear. Utilizing data from the 2007–2018 National Health and Nutrition Examination Survey (NHANES), this study investigates the relationship between multiple carotenoid subtypes and DN, aiming to inform exploratory insights for potential preventive strategies.

**Methods:**

This cross-sectional analysis utilized NHANES 2007–2018 data with a multistage stratified sampling design. After a four-stage screening process, baseline participants were selected by excluding individuals with lacking diabetes-related data, pregnant women, and those with incomplete carotenoid intake or DN diagnostic records. Model 1 included 25,483 participants, while Model 2 comprised retained 13,271 participants after further adjustment for covariates (demographic characteristics, lifestyle factors, clinical indicators, and socioeconomic parameters). Dietary intake of six carotenoid subtypes (*α-carotene, β-carotene, β-cryptoxanthin, Lycopene, Lutein, Zeaxanthin*) was calculated using as the mean of two standardized 24-h dietary recalls. Type 2 diabetes mellitus (T2DM) was diagnosed based on fasting plasma glucose (≥126 mg/dL), HbA1c (≥6.5%), hypoglycemic medication use, or clinical diagnosis. DN was defined as a urinary albumin-to-creatinine ratio (UACR) ≥ 30 mg/g or estimated glomerular filtration rate (eGFR) < 60 mL/min/1.73 m^2^ in T2DM patients. Statistical analyses employed weighted multivariable logistic regression using the R survey package, adjusting for sampling design and covariates. Associations were quantified as odds ratios (ORs per 10 μg/d carotenoid intake) with Benjamini-Hochberg correction for multiple comparisons. The significance threshold was set at *α* = 0.05.

**Results:**

This NHANES-based study revealed nonlinear associations between dietary carotenoids and diabetic nephropathy (DN) risk, with notable gender-and ethnicity-specific gender/ethnicity-specific effects. Univariate analysis demonstrated elevated DN risk with higher β-cryptoxanthin intake (OR = 1.413, 95% CI), though significance was attenuated dissipated after multivariable adjustment, suggesting confounding mediation by obesity and hypertension. Multivariable models identified threshold effects: the low *α*-carotene intake group exhibited a 75% reduced risk (OR = 0.25, 95% CI), while the high-intake group showed a paradoxical risk elevation (OR = 2.24, 95% CI). β-Carotene displayed gender-divergent effects, with the highest tertile significantly reducing risk in males by 43% (OR = 0.57, 95% CI). Interaction models highlighted intensified *α*-carotene protection in males and Non-Hispanic White (OR = 0.27–0.29), whereas lycopene correlated with a fivefold increased DN risk in Non-Hispanic Black (OR = 4.99, 95% CI). Cardinal risk factors included advanced age (OR = 1.06/year), obesity (OR = 1.07/BMI unit), male sex (OR = 3.05), and hypertension (OR = 4.7), while higher education (OR = 0.72) and moderate alcohol consumption exerted inversely associated effects. These findings underscore the necessity of integrating nutrient thresholds (e.g., *α*-carotene optimization) and social determinants into DN prevention, though racial subgroup analyses warrant validation through prospective cohorts due to sample size constraints.

**Conclusion:**

Clinicians managing DN should: Prioritize Consider *α*-carotene’s biphasic dose–response relationship, prioritizing intake within the optimal dosage window; Utilize Leverage β-carotene’s gender-specific benefits for male patients; Exercise caution in consider exploringing lycopene-rich dietary interventions diets for African American populations. Public health initiatives should incorporate nutrient-gender-ethnicity triadic assessments into DN education programs to advance precision nutrition guidelines.

## 1 Introduction

Diabetic Nephropathy (DN) is one of the most serious microvascular complications of diabetes mellitus and is characterized by a progressive decline in renal function that eventually leads to End-Stage Renal Disease (ESRD) (1). Epidemiologic studies have shown that 30–50% of the U. S. diabetic population will develop DN as a major causative factor for end-stage renal disease (2, 3).

The pathological progression of diabetic nephropathy (DN) is synergistically driven by three major metabolic disturbances: imbalance of glucose homeostasis triggers glomerular basement membrane thickening and fibrosis through glycosylation end-product production, abnormal polyol metabolism, and activation of protein kinase C pathway; Abnormal blood pressure regulation: accelerated glomerulosclerosis and decreased filtration function through high intraglomerular pressure and activation of the renin-angiotensin system; disorders of lipid metabolism through lipotoxicity-mediated oxidative stress, TLR4/NF-κB inflammatory signaling and insulin resistance. Disorders of adipose metabolism, on the other hand, exacerbate renal injury through lipotoxicity-mediated oxidative stress, TLR4/NF-κB inflammatory signaling, and insulin resistance-associated renal hemodynamic alterations (4–6).

Recent studies have confirmed that dietary components are directly involved in the pathologic process of diabetic microangiopathy through molecular pathways such as epigenetic modification, oxidative stress regulation, and intestinal flora-mediated pathways (7–9). Exploring the dose-effect relationship between specific nutrients and the development of DN has become an important research direction in the field of nutritional epidemiology of chronic diseases.

Carotenoids are a class of bioactive compounds widely found in plant foods such as vegetables and fruits, and their antioxidant and anti-inflammatory properties have been confirmed by several studies (10, 11). Epidemiologic studies suggest that dietary carotenoid intake is negatively associated with the risk of cardiovascular disease and specific cancers (12). Oxidative stress and chronic inflammatory response have been shown to be involved in the pathophysiological process of diabetic nephropathy (DN). Animal experiments have shown that in db/db mice (a model of type 2 diabetes), dietary supplementation with 0.004% siphon xanthin attenuated renal morphological alterations (including glomerular basement membrane thickening and tethered membrane matrix dilation) although it did not significantly change plasma creatinine and urinary albumin levels and improved lipid metabolism by restoring fatty acid β oxidation-associated protein mRNA expression in skeletal muscle (13); and astaxanthin long-term intervention (12 weeks) significantly reduced blood glucose levels, inhibited the elevation of urinary albumin and 8-OHdG in diabetic mice (decreased compared with the untreated group, respectively), and delayed the progression of DN through antioxidant effects as evidenced by the reduction in the ratio of the tethered membrane area/glomerular area (improved compared with the untreated group) and by the decrease of the number of glomerular 8-OHdG-immunoreactive cells (14), suggesting that carotenoids may regulate the risk of DN development through the above mentioned mechanisms, suggesting that carotenoids may modulate the risk of DN development. However, there is no clear consensus on the association between dietary carotenoids and the risk of DN development.

Previous carotenoid-DN association studies were mostly limited by insufficient sample size (e.g., Ref. (15), *n* = 136) and lack of evidence from large prospective cohorts. Methodologically, most studies have not corrected for covariate interference between carotenoid subclasses (e.g., high correlation between β-carotene and *α*-carotene) and have not covered a wider range of populations through weighting analyses to enhance extrapolation of results. In this study, we systematically assessed the association between dietary carotenoid subclasses (α-carotene, β-carotene, β-cryptoxanthin, lycopene, and lutein-zeaxanthin complex) and diabetic nephropathy (DN) based on cross-sectional data from the National Health and Nutrition Examination Survey (NHANES) from 2007 to 2018. The study included adult patients with diabetic nephropathy as the target population, quantified the intake of six carotenoids using the 24-h dietary review method, and the diagnosis of DN was based on the urinary albumin/creatinine ratio (UACR) and glomerular filtration rate (eGFR) criteria (16).

This study included a sample of 25,484 adults for association exploration based on the NHANES database, and included a sample of Example 13,272 adults for refined analysis after adjusting for covariates. The interaction effect of total carotenoids with ethnicity was explored, as well as how gender differences play a role in the interaction between carotenoids and DN, revealing the potential moderating role of individual demographic characteristics on the carotenoid-DN association. By weighted multivariate logistic regression modeling and correcting for confounding factors such as demographic characteristics, metabolic indicators and lifestyle, the independent and interactive effects of carotenoid subclasses and DN risk were resolved at a multidimensional level for the first time. The study design overcame the shortcomings of previous under-represented samples and provided epidemiological evidence for the association between carotenoid bioavailability and the pathogenesis of DN.

## 2 Research methodology

### 2.1 Study population

The data for this study were obtained from the National Health and Nutrition Examination Survey (NHANES) for six consecutive survey cycles from 2007 to 2018. NHANES, as a national multistage stratified sample study under the auspices of the National Center for Health Statistics (NCHS), collects data on the health and nutritional status of the U. S. population by means of a system of laboratory tests, physical examinations, and structured questionnaires, and all participants have signed a written informed consent form. All participants signed a written informed consent. Raw data were publicly available through an official platform^1^.

A total of 59,842 baseline participants were included in the study, and the final sample was determined through a four-stage screening process. The first stage excluded 2,466 individuals with no recorded diabetes-related data (see section 2.3 for specific exclusion criteria), obtaining 57,376 study participants; the second stage excluded 372 individuals during pregnancy, leaving 57,004; and the third stage excluded 14,337 individuals with missing data on carotenoid intake and 17,184 who did not satisfy the conditions of the diabetic nephropathy modeling, forming the Model 1 baseline sample (*n* = 25,483).

**Figure 1:**
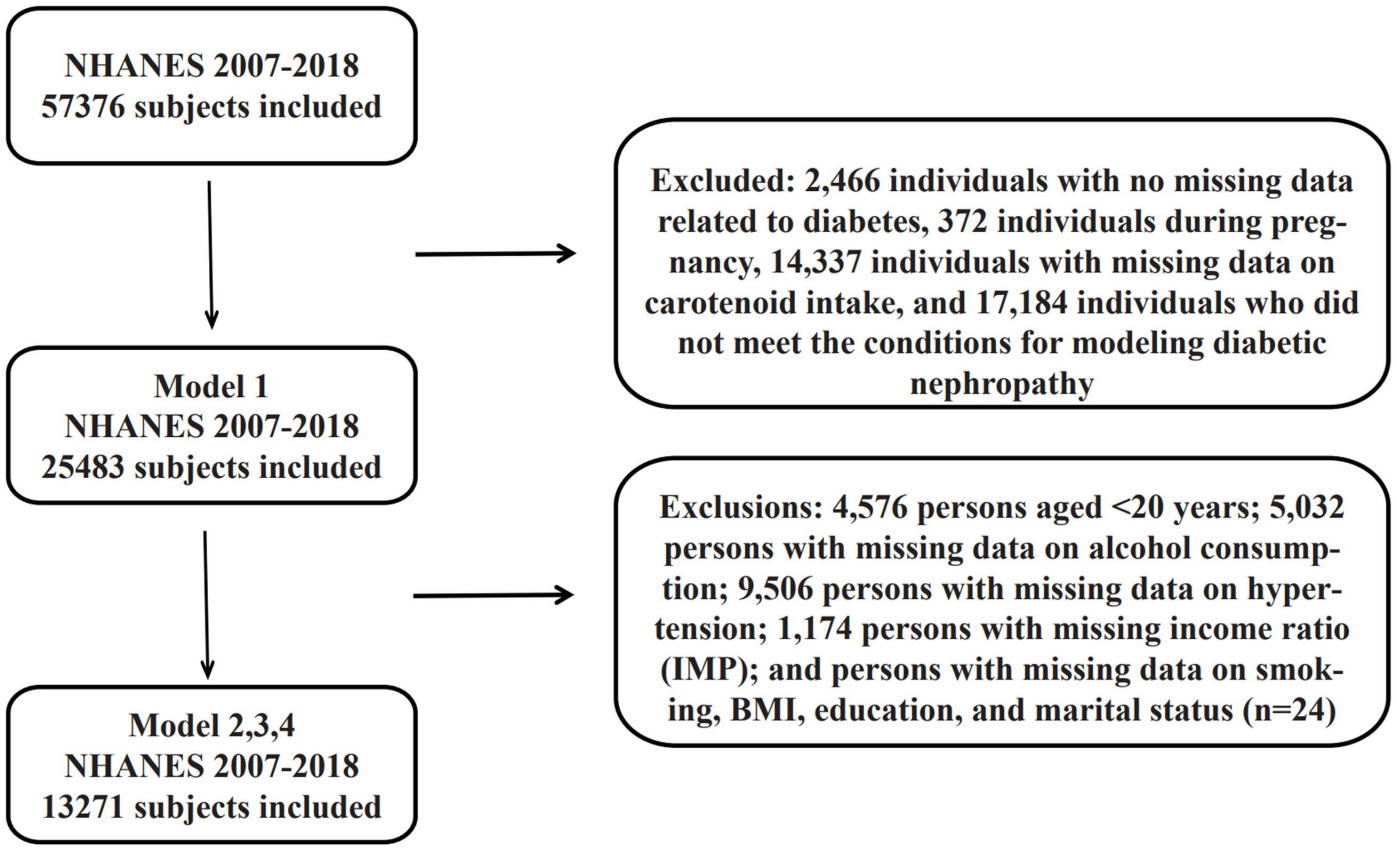
Flowchart of population inclusion and screening criteria.

Model 2 was adjusted for covariates based on Model 1 (see Section 2.4 for adjustment criteria), and further excluded through a systematic screening process: (i) 4,576 patients aged <20 years; (ii) 5,032 patients with missing data on alcohol consumption; (iii) 9,506 patients with missing data on hypertension; (iv) 1,174 patients with missing income ratio (IMP); and (v) patients with missing data on smoking, BMI, education and marital status (*n* = 24). (*n* = 24); ultimately 13,271 samples were obtained that met all covariate completeness requirements. All data cleansing processes were conducted through multiple verification mechanisms to ensure the accuracy of the screening logic.

### 2.2 Dietary carotenoids

Dietary intake data for NHANES participants were collected through two standardized 24-h dietary recall methods. The first data collection was completed on-site at the Mobile Examination Center (MEC) by a NHANES-trained and certified dietitian, and the second recall was conducted by telephone within 3–10 days of the initial interview (median interval 6 days). Both dietary recalls were conducted using the USDA Automated Multiple-Pass Method (AMPA) to systematically enhance the completeness and accuracy of food recall.

This study focused on the average daily intake (in μg/day) of six carotenoids including: *α*-carotene, β-carotene, β-cryptoxanthin, lycopene, lutein and zeaxanthin. All intake data were calculated based on the average of two dietary reviews to minimize possible bias from a single review.

### 2.3 Health assessment and diagnosis of diabetic nephropathy

Health assessment process: all health indicators were completed with standardized testing at specialized Mobile Examination Centers (MEC) across the United States.

Type 2 diabetes mellitus (T2DM) definition: T2DM was diagnosed by meeting any of the following conditions (17):

Self-reported diagnosis of diabetes by a clinician.
Fasting plasma glucose (FPG) ≥ 126 mg/dL.
Glycosylated hemoglobin (HbA1c) ≥ 6.5%.
Currently using insulin or oral hypoglycemic agents.

Diagnostic criteria for Diabetic Nephropathy (DN): Patients with T2DM are diagnosed with DN if they fulfill any of the following criteria (18):

Urine albumin-to-creatinine ratio (UACR) ≥ 30 mg/g.
Estimated glomerular filtration rate (eGFR) < 60 mL/min/1.73 m^2^ (based on the CKD-EPI formula).

### 2.4 Covariates

Covariates were collected through standardized questionnaires, physical examination and laboratory tests including:

Demographic characteristics: age, gender, race/ethnicity (Mexican, other Hispanic, Non-Hispanic White, Non-Hispanic Black, and other races), marital status (married, widowed, divorced, separated, unmarried, or cohabiting) and education level (did not complete 9th grade, 9th-11th grade, high school graduate, college incomplete, or college graduate and above).

#### 2.4.1 Behavioral factors

Smoking status: never smoked (<100 lifetime cigarettes), previous smoker (≥100 cigarettes and not a current smoker), or current smoker (≥100 cigarettes and a current smoker).

Alcohol consumption status (19): no alcohol consumption (<12 lifetime drinks), moderate alcohol consumption (≤1 standard drink/day for women and ≤2 standard drinks/day for men), or excessive alcohol consumption (>1 standard drink/day for women and >2 standard drinks/day for men; 1 standard drink = 14 g of pure alcohol).

#### 2.4.2 Clinical indicators

Hypertension (20): defined as systolic blood pressure ≥140 mmHg, diastolic blood pressure ≥90 mmHg, or self-reported having been diagnosed with hypertension by a physician.

Body mass index (BMI) (21): calculated as weight (kg)/height (m^2^).

### 2.5 Statistical analysis

#### 2.5.1 Data pre-processing

This study strictly followed the Centers for Disease Control and Prevention (CDC) National Health and Nutrition Examination Survey (NHANES) data analysis guidelines. To address the complex sampling design features, data were weighted using stratified cluster sampling weights, and standard error correction was applied by Taylor linearization method (22). Dietary carotenoid intake was processed as follows: participants were categorized into a zero intake group based on intake and a non-zero intake group, which was divided into four equally spaced exposure classes using the quartile method.

#### 2.5.2 Assessment of normality

A two-dimensional approach was used in this study to assess the distributional characteristics of continuous variables (Supplementary Figure 1):

a Lilliefors modified Kolmogorov–Smirnov test.
b Anderson-Darling test.

#### 2.5.3 Baseline characterization

A two-column control table was used to present weighted and unweighted baseline characteristics. Continuous variables were described using a joint description: mean ± standard deviation (Mean ± SD) jointly reported with median (interquartile range, Median [Q1, Q3]). Categorical variables were expressed as weighted percentages (95% confidence interval).

#### 2.5.4 Methods of comparison between groups

The test of difference strategy was selected based on the distributional characteristics of the variables:

Normal continuous variables: design-based weighted *t*-tests.
Non-normal continuous variables: weighted Mann–Whitney U test based on rank order was applied (svyranktest() function implementation).
Categorical variables: apply Rao-Scott modified chi-square test, switch to exact test when cell expected frequency < 5.

#### 2.5.5 Multivariate regression modeling

The model was constructed using an incremental strategy:

Model 1 (crude model): univariate analysis, only carotenoid subclasses were included.
Model 2 (fully adjusted model): adjusted for demographics (age, sex, race, education, marital status, poverty-to-income ratio), lifestyle (smoking status: current/previous/not; alcohol use: heavy drinking/moderate/withdrawal) and clinical indicators (hypertension diagnosis, body mass index).
Model 3 (interaction effect model): assessing the product interaction term between the six carotenoids and race.
Model 4 (subgroup modeling): exploring the effect modifier role of gender on the carotenoid-diabetic nephropathy association.

#### 2.5.6 Multicollinearity control

Assessed bi-dimensionally by Pearson correlation coefficient matrix. The Pearson correlation coefficients between carotenoid subclasses were all <0.7 (Supplementary Figure 2), satisfying the model covariance control criteria.

#### 2.5.7 Reporting of effect sizes

Odds Ratio (OR) and its 95% confidence interval corresponding to each 10 μg/d increment of carotenoid intake were used as main effect indicators.

#### 2.5.8 Implementation of statistical analysis

Weighted analyses were performed using the survey extension package (version 4.2–1) for the R language (version 4.4.1). Hypothesis testing was performed using two-sided tests, with the significance threshold set at *α* = 0.05. For multiple comparisons of the six carotenoid subclasses, the Benjamini-Hochberg method was applied for false discovery rate control.

## 3 Results

### 3.1 Participant characteristics

#### 3.1.1 Statistical methods and data description

In this study, baseline characteristics of patients with diabetic nephropathy and non-diabetic nephropathy were analyzed in comparison to each other, presenting unweighted samples and weighted-adjusted results, respectively (Table 1). The data are presented below in a standardized format:

(1) Sample characteristics
  - Unweighted sample: 816 cases in diabetic nephropathy group vs. 12,455 cases in non-diabetic nephropathy group.
  - Weighted sample (considering complex sampling design weights): 4,104,708.8 weighted individuals in the diabetic nephropathy group vs. 98,237,592.2 weighted individuals in the non-diabetic nephropathy group.
(2) Comparison of continuous variables (mean ± standard deviation)
  Age: significant difference between unweighted groups (65.6 ± 13.1 vs. 46.9 ± 16.9, *p* < 0.001), maintained after weighting (67.0 ± 13.1 vs. 46.0 ± 16.9, *p* < 0.001).
  BMI: both groups showed higher in the diabetic nephropathy group (unweighted 32.1 ± 7.3 vs. 29.1 ± 6.9; weighted 31.7 ± 7.3 vs. 27.8 ± 6.9, both *p* < 0.001).
  PIR (household income poverty ratio): statistically different between unweighted groups (2.4 ± 1.5 vs. 2.7 ± 1.7, *p* < 0.001), significant but directionally reversed after weighting (2.57 ± 1.5 vs. 3.30 ± 1.7, *p* < 0.001).
(3) Distribution of categorical variables (chi-square test)
  Nutritional intake: carotenoid intake stratification of *α*-carotene, β-carotene, and β-cryptoxanthin were not significantly different between the two groups (*p* > 0.05).
  Metabolic risk factors: prevalence of hypertension was significantly higher in the diabetic nephropathy group (unweighted 80.6% vs. 37.2%; weighted 79.9% vs. 33.6%, both *p* < 0.001).
  Lifestyle: alcohol abuse was lower in the diabetic nephropathy group (unweighted 25.1% vs. 44.0%) and current smoking was lower (14.2% vs. 20.0%), both *p* < 0.001.
  Sociodemographics: weighted analysis showed racial differences remained significant (*p* = 0.002), with a higher percentage of Non-Hispanic Black participants in the diabetic nephropathy group (13.8% vs. 9.6%).
(4) Description of statistical methods

**Table 1:** Data from 13,271 participants, grouped by diabetic nephropathy.

	Total *N* = 13,271	No diabetic nephropathy *N* = 12,455	Diabetic nephropathy *N* = 816	*p* value
α-carotene	81.34% Zero values; Nonzero average: 72.63	85.55% Zero values; Nonzero mean: 73.72	17.16% Zero values; Nonzero mean: 54.51	**0.26**
β-carotene	45.84% Zero values; Nonzero mean: 144.51	45.66%Zero values; Nonzero mean: 146.03	48.53%Zero values; Nonzero mean: 120.03	**0.121**
β-cryptoxanthin	79% Zero values; Nonzero mean: 24.73	82.79% Zero values; Nonzero mean: 24.43	21.08% Zero values; Nonzero mean: 29.32	**0.712**
lycopene	91.33% Zero values; Nonzero mean: 2558.28	96.83% Zero values; Nonzero mean: 2591.79	7.35% Zero values; Nonzero mean: 1949.45	**0.163**
Lutein + Zeaxanthin	47.79% Zero values; Nonzero mean: 90.01	49.5% Zero values; Nonzero mean: 103.16	47.68% Zero values; Nonzero mean: 89.17	**0.326**
Age	47.0 [33.0;62.0]	46.0 [32.0; 60.0]	67.5 [58.0; 78.0]	**<0.001**
Gender				**<0.001**
Male	6,552 (49.40%)	6,028 (48.40%)	524 (64.20%)	
Female	6,719 (50.60%)	6,427 (51.60%)	292 (35.80%)	
Hypertension				**<0.001**
Normal	7,979 (60.10%)	7,821 (62.80%)	158 (19.40%)	
Hypertension	5,292 (39.90%)	4,634 (37.20%)	658 (80.60%)	
Smoking status				**<0.001**
Never smoke	7,617 (57.40%)	7,220 (58.00%)	397 (48.70%)	
History of smoking	3,049 (23.00%)	2,746 (22.00%)	303 (37.10%)	
Currently smoke	2,605 (19.60%)	2,489 (20.00%)	116 (14.20%)	
Drinking status				**<0.001**
Never drink	7,617 (57.40%)	7,220 (58.00%)	397 (48.70%)	
Drink moderately	3,049 (23.00%)	2,746 (22.00%)	303 (37.10%)	
Alcohol abuse	2,605 (19.60%)	2,489 (20.00%)	116 (14.20%)	
BMI				**<0.001**
≤24.9	3,755 (28.30%)	3,636 (29.20%)	119 (14.60%)	
25–29.9	4,383 (33.00%)	4,142 (33.30%)	241 (29.50%)	
≥30	5,133 (38.70%)	4,677 (37.60%)	456 (55.90%)	
PIR				**<0.001**
≥3.55	4,469 (33.70%)	4,283 (34.40%)	186 (22.80%)	
≥1.3–3.5	4,986 (37.60%)	4,611 (37.00%)	375 (46.00%)	
01.3	3,816 (28.80%)	3,561 (28.60%)	255 (31.20%)	
Race				**<0.001**
Mexican American	1827 (13.80%)	1708 (13.70%)	119 (14.60%)	
Other Hispanic	1,309 (9.86%)	1,243 (9.98%)	66 (8.09%)	
NonHispanic White	5,850 (44.10%)	5,478 (44.00%)	372 (45.60%)	
NonHispanic Black	2,675 (20.20%)	2,479 (19.90%)	196 (24.00%)	
Other Race	1,610 (12.10%)	1,547 (12.40%)	63 (7.72%)	
Educational level				**<0.001**
Less than 9th grade	904 (6.81%)	790 (6.34%)	114 (14.00%)	
9-11th grade	1,497 (11.30%)	1,380 (11.10%)	117 (14.30%)	
High school graduate	2,901 (21.90%)	2,708 (21.70%)	193 (23.70%)	
Some college	4,283 (32.30%)	4,046 (32.50%)	237 (29.00%)	
College graduate or above	3,686 (27.80%)	3,531 (28.40%)	155 (19.00%)	
Marital status				**<0.001**
Married	6,843 (51.60%)	6,386 (51.30%)	457 (56.00%)	
Widowed	815 (6.14%)	684 (5.49%)	131 (16.10%)	
Divorced	1,444 (10.90%)	1,330 (10.70%)	114 (14.00%)	
Separated	403 (3.04%)	379 (3.04%)	24 (2.94%)	
Never married	2,626 (19.80%)	2,559 (20.50%)	67 (8.21%)	
Living with partner	1,140 (8.59%)	1,117 (8.97%)	23 (2.82%)	

High zero - value ratios in carotenoids; no carotenoid level difference. Differences in demographics, etc. Diabetic nephropathy patients more likely male, older, etc. Bold values indicate statistically significant differences (p < 0.05) between the “without diabetic nephropathy” and “Diabetic Nephropathy” groups, as determined by Two Sample t-test, Wilcoxon rank sum test, or Pearson’s Chi-squared test. Non-bold values represent comparisons where no statistically significant difference was observed (p ≥ 0.05). “N” denotes the sample size (unweighted or weighted, as specified). Percentages (%) are presented for categorical variables, while mean (SD) or median (Q1, Q3) are reported for continuous variables. P3 refers to the p-value for the comparison between groups.

Independent samples *t*-test or Wilcoxon rank sum test was used for continuous variables and chi-square test for categorical variables. Weighted analyses used NHANES complex sampling weights to more accurately reflect U. S. population characteristics. All tests were two-sided with *α* = 0.05.

In this study, the sample selection bias was effectively corrected by weighted analysis, which confirmed that patients with diabetic nephropathy were characterized by more significant metabolic risk factors such as advanced age, obesity, and hypertension, as well as significant differences in social determinants such as education level and marital status.

### 3.2 Model 1: one-way logistic regression analysis of dietary carotenoid intake and risk of diabetic nephropathy

In this study, the crude association between different levels of dietary carotenoid intake and the risk of diabetic nephropathy (using non-consumers as the reference group, REF) was assessed by a one-way (unadjusted for confounders) logistic regression model. The results were as follows:

1 *α*-carotene (α-carotene)

**Figure 2:**
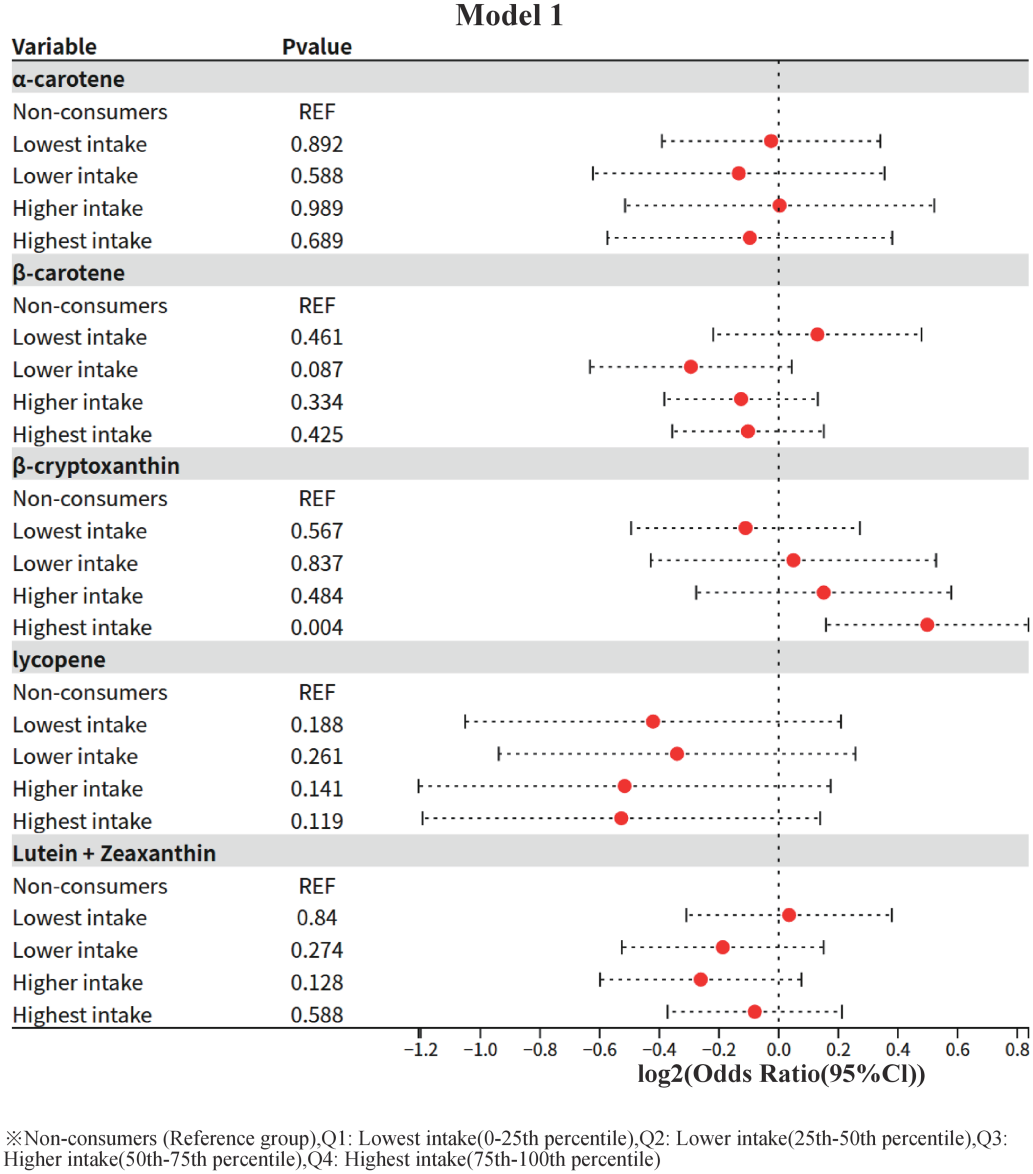
Model 1: Univariate logistic regression.

The ORs of all intake groups (lowest, lower, higher, and highest) were not statistically significant (all *p* > 0.05), suggesting that α-carotene intake is not significantly associated with the risk of diabetic nephropathy.

2 β-carotene (β-carotene)

The OR value for the lower intake group (Lower intake) showed a potentially protective trend (OR = 0.816, 95% CI: 0.645–1.031) but did not reach statistical significance (*p* = 0.087). The ORs of the other intake groups were close to 1 and non-significant (OR = 0.931 for the highest intake group, *p* = 0.425).

3 β-cryptoxanthin (β-cryptoxanthin)

The risk of diabetic nephropathy was significantly higher in the highest intake group (OR = 1.413, 95% CI: 1.116–1.787, *p* = 0.004), whereas the OR values of the other intake groups (lowest, lower, higher) were not statistically significant (*p* > 0.05). This result suggests that high-dose β-cryptoxanthin intake may be associated with an increased risk of diabetic nephropathy without adjusting for confounders.

4 Lycopene

ORs of all intake groups did not show statistical significance (*p* > 0.05), and the protective trend did not reach a significant level in the lowest intake group (OR = 0.747, *p* = 0.188) and the highest intake group (OR = 0.694, *p* = 0.119).

Lutein + zeaxanthin (Lutein + Zeaxanthin)

The OR values of all intake groups were close to 1 and not statistically significant (*p* > 0.05), suggesting that this nutrient is not significantly associated with the risk of diabetic nephropathy.

### 3.3 Model 2: results of multifactorial logistic regression analysis

In this study, the independent associations of dietary carotenoids and other covariates with the risk of diabetic nephropathy were assessed by multifactorial logistic regression modeling (adjusting for demographic, clinical and lifestyle confounders). The results were as follows (using non-consumers as the baseline group reference, REF):

1 Dietary carotenoid intake.
  Alpha-carotene (*α*-carotene)

**Figure 3:**
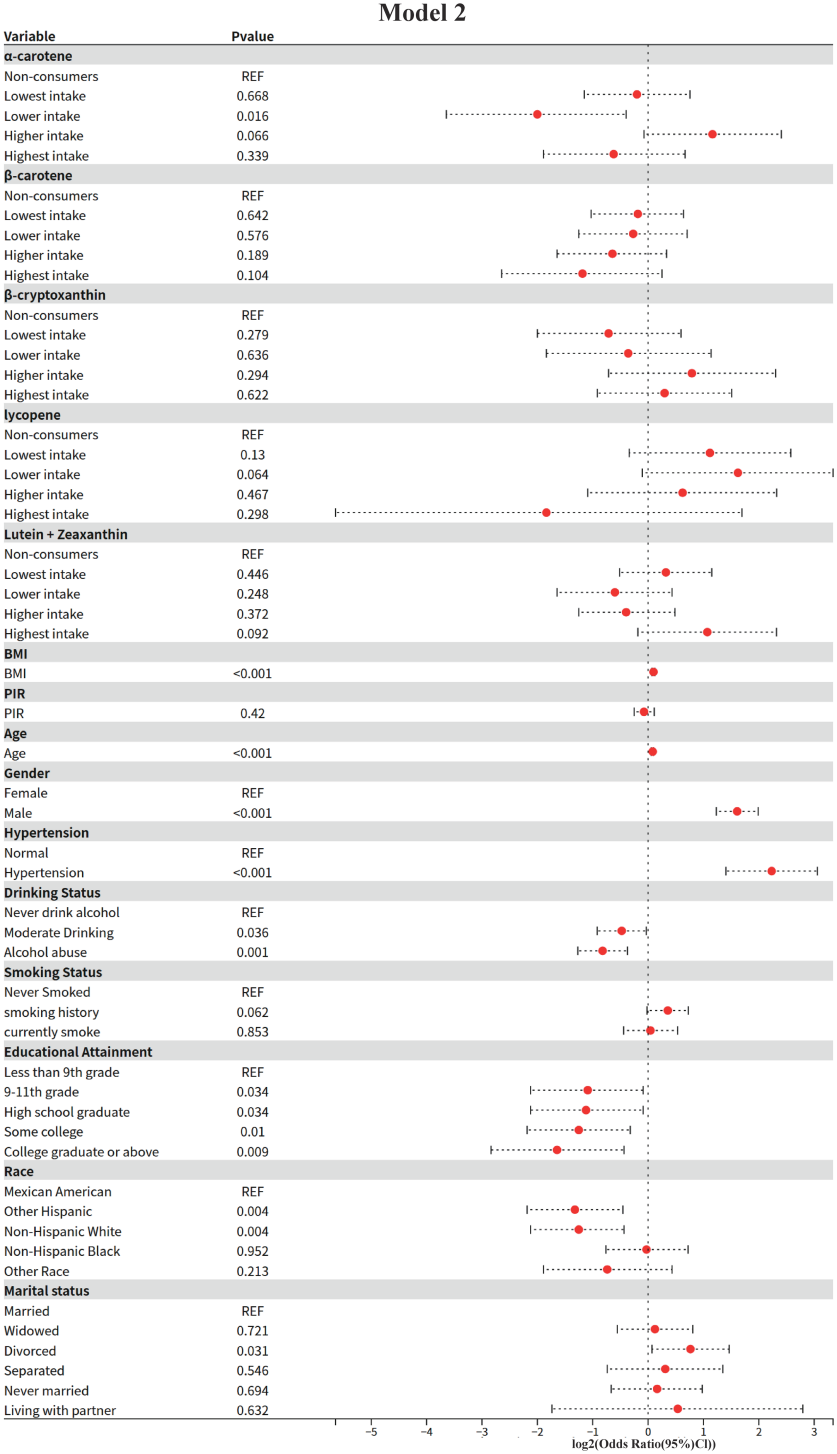
Model 2: Multivariate logistic regression.

Lower intake group (Lower intake): significantly lower risk of diabetic nephropathy (OR = 0.25, 95% CI: 0.08–0.76, *p* = 0.016).

Higher intake group: non-significantly higher risk (OR = 2.24, 95% CI: 0.95–5.30, *p* = 0.066).

Other intake groups: neither the lowest (OR = 0.87, *p* = 0.668) nor the highest (OR = 0.65, *p* = 0.339) groups were statistically significant.

Beta-carotene (β-carotene)

ORs were not statistically significant (*p* > 0.05) in all intake groups, but the highest intake group showed a potential protective trend (OR = 0.44, 95% CI: 0.16–1.19, *p* = 0.104).

Beta-cryptoxanthin (β-cryptoxanthin)

ORs were not statistically significant for all intake groups (*p* > 0.05), with the highest intake group not reaching a significant level of risk (OR = 1.23, 95% CI: 0.53–2.85, *p* = 0.622).

Key change: Significance disappeared in the highest intake group compared to the univariate model (Model I), suggesting that the risk may be mediated by confounding factors (e.g., BMI, hypertension, etc.).

Lycopene

Lower intake group: borderline elevated risk (OR = 3.08, 95% CI: 0.93–10.14, *p* = 0.064).

Other intake groups: no significant association (*p* > 0.05).

Lutein + Zeaxanthin (Lutein + Zeaxanthin)

All intake groups were not statistically significant (*p* > 0.05), but there was a trend toward a potentially higher risk in the highest intake group (OR = 2.10, 95% CI: 0.88–4.99, *p* = 0.092).

2 Demographic and clinical covariates
  Core risk factors

BMI: For each 1-unit increase, the risk of diabetic nephropathy was elevated by 7% (OR = 1.07, 95% CI: 1.05–1.09, and *p* < 0.001).

Age: 6% higher risk per 1-year increase (OR = 1.06, 95% CI: 1.04–1.07, *p* < 0.001).

Sex: men had a 3.05 times higher risk than women (95% CI: 2.35–3.97, *p* < 0.001).

Hypertension: the risk was 4.7 times higher in hypertensives than in non-hypertensives (95% CI: 2.65–8.33, *p* < 0.001).

Protective factors

Educational level: risk was lower with higher education compared with “less than 9th grade” (e.g., “college and above”) (OR = 0.32, 95% CI: 0.14–0.74, *p* = 0.009).

Race: Non-Hispanic White (OR = 0.42, *p* = 0.004) and other Hispanics (OR = 0.40, *p* = 0.004) had significantly lower risks than Mexicans.

Lifestyle and marital status

Alcohol consumption: both moderate alcohol consumption (OR = 0.72, *p* = 0.036) and alcohol abuse (OR = 0.57, *p* = 0.001) showed protective effects.

Smoking: current smokers had no significant change in risk (OR = 1.03, *p* = 0.853), but those with a history of smoking had a borderline elevated risk (OR = 1.28, *p* = 0.062).

Marital status: divorcees had a significantly higher risk (OR = 1.70, 95% CI: 1.05–2.76, *p* = 0.031).

### 3.4 Model 3: interaction of gender, race and carotenoids

The interaction between carotenoids and demographic variables was systematically evaluated based on Model 2, and the specific process was as follows: firstly, the extended formula containing the interaction term was dynamically constructed based on the main effects model, and the generalized linear model weighted by complex survey design was fitted through the svyglm function; subsequently, the overall significance of the interaction term was tested by applying the regTermTest (likelihood ratio test, *α* = 0.05), and the interaction term was further parameterized by calculating the ratio (OR) and 95% confidence intervals using a t-distribution to adjust the degrees of freedom. Interactions were further carried out for parameter estimation, t-distribution was used to adjust the degrees of freedom when calculating the ratio of ratios (OR) and 95% confidence intervals, in which the standard errors were calculated by weighting the model covariance matrix; the final results were implemented as a double-testing strategy, and the Wald test was used to assess the specific effects of each interaction term, with all the *p*-values corrected for Bonferroni, under the premise that the overall test was passed.

1 *α*-Carotene
  Sex interaction: significant (overall *p* = 0.029).
  Higher intake in men: OR = 0.272 (95% CI: 0.088–0.842, *p* = 0.025), implying that the effect for alpha-carotene was only one-fourth as large in men compared to women.
  Race interaction: borderline significant (overall *p* = 0.05).
  Non-Hispanic White minimum intake: OR = 0.291 (95% CI: 0.096–0.88, *p* = 0.03), implying that compared to Mexican Americans, the Non-Hispanic White minimum intake had only 30% of the effect for α-carotene effect was only 30%.
  No significant associations were found for any of the other races (Mexican-American, Non-Hispanic Black, etc.), and some subgroup confidence intervals were extremely wide (e.g., highest intake for other races CI: 0.043–3.512), reflecting inadequate sample sizes.
2 β-carotene
  Sex interaction: not significant (overall *p* = 0.61).
  Race interaction: not significant (overall *p* = 0.999).
3 β-Cryptoxanthin
  Sex interaction: not significant (overall *p* = 0.576).
  Direction of ORs was not consistent across intake levels for males (lowest intake OR = 1.674 vs. higher intake OR = 0.624), but neither was statistically significant.
  Race interaction: highly significant (overall *p* < 0.001).
  Other Hispanic next lowest intake: missing data.
  Non-Hispanic Black highest intake: near significance (OR = 0.474, *p* = 0.166) but confidence interval contains 1 (CI: 0.162–1.389).
4 Lycopene
  Sex interaction: not significant (overall *p* = 0.216).
  Maximum intake for males: abnormally high OR = 4.586 (CI: 0.375–56.099, *p* = 0.226), with very wide confidence intervals, suggesting small sample bias.
  Race interaction: highly significant (overall *p* < 0.001).
  Other Hispanics: significant missing data, some confidence intervals spanning more than 30-fold (e.g., highest intake CI: 0.034–36.72).
  Non-Hispanic Black higher intake: OR = 4.993 (CI: 0.759–32.859, *p* = 0.092), potentially high risk but lack of precision.
5 Lutein + zeaxanthin
  Sex interaction: not significant (overall *p* = 0.299).
  Race interaction: not significant (overall *p* = 0.719).
  Higher intake in Non-Hispanic White: close to significant (OR = 0.414, *p* = 0.047), but CI upper limit close to 1 (0.174–0.987), need to be interpreted with caution.

### 3.5 Model 4: gender subgroup analysis

The effect modifying role of gender on the carotenoid-disease association was systematically assessed based on Model 2. The Kish approximation formula was first applied to calculate the effective sample size for data quality control (threshold n_eff ≥ 50); weighted logistic regression models were subsequently fitted; to address the problem of multiple testing, the rate of false discovery was controlled within subgroups using the Benjamini-Hochberg method (p.adjust (method = “BH”)), and for between-groups two-by-two comparisons using the Bonferroni correction; analysis of between-group heterogeneity was achieved through a two-stage process: (1) assessing gender main effects based on the global Wald test, and (2) generating all gender-combined pairs using combn, and calculating OR differences between subgroups and their Bonferroni-corrected *p*-values via svyby combined with svyglm.

#### 3.5.1 Key findings (gender stratification)

1 Shared risk and protective factors

BMI and age: both sexes showed significant positive associations (men: OR = 1.08/year, women: OR = 1.07/year, both *p* < 0.001).

Hypertension: risk effect was significantly higher in women than men (women OR = 4.7 vs. men OR = 2.11, both p < 0.001).

Race protection: Non-Hispanic White were all at lower risk than Mexicans (male OR = 0.59, female OR = 0.42, *p* ≤ 0.033).

2 Gender-specific associations

Economic income (PIR): 15% lower risk in higher income groups for men only (OR = 0.85, *p* = 0.006), no association for women (OR = 0.95, *p* = 0.594).

Educational attainment: only females with a bachelor’s degree or higher showed a potential protective effect (OR = 0.32, *p* = 0.057), no significant association in males.

#### 3.5.2 Behavioral factors

Alcohol consumption: moderate alcohol consumption was potentially protective in women (OR = 0.54, *p* = 0.061), no association in men.

Smoking: trend toward higher risk for smoking history in females (OR = 1.66, *p* = 0.061), no association in males.

3 Differences in carotenoid effects.

Beta-carotene: highest intake significantly reduced risk by 43% only in men (OR = 0.57, *p* = 0.032), no association in women.

*α*-carotene: potential protection at next-lowest intake (OR = 0.25, p = 0.061) and elevated risk at higher intake (OR = 2.24, *p* = 0.168) in women, suggesting a J-curve; no association in men.

Beta-cryptoxanthin: doubled risk at next-lowest/highest intake only in men (OR = 2.03–2.16, *p* < 0.05), no significant association in women.

## 4 Discussion

**Figure 4:**
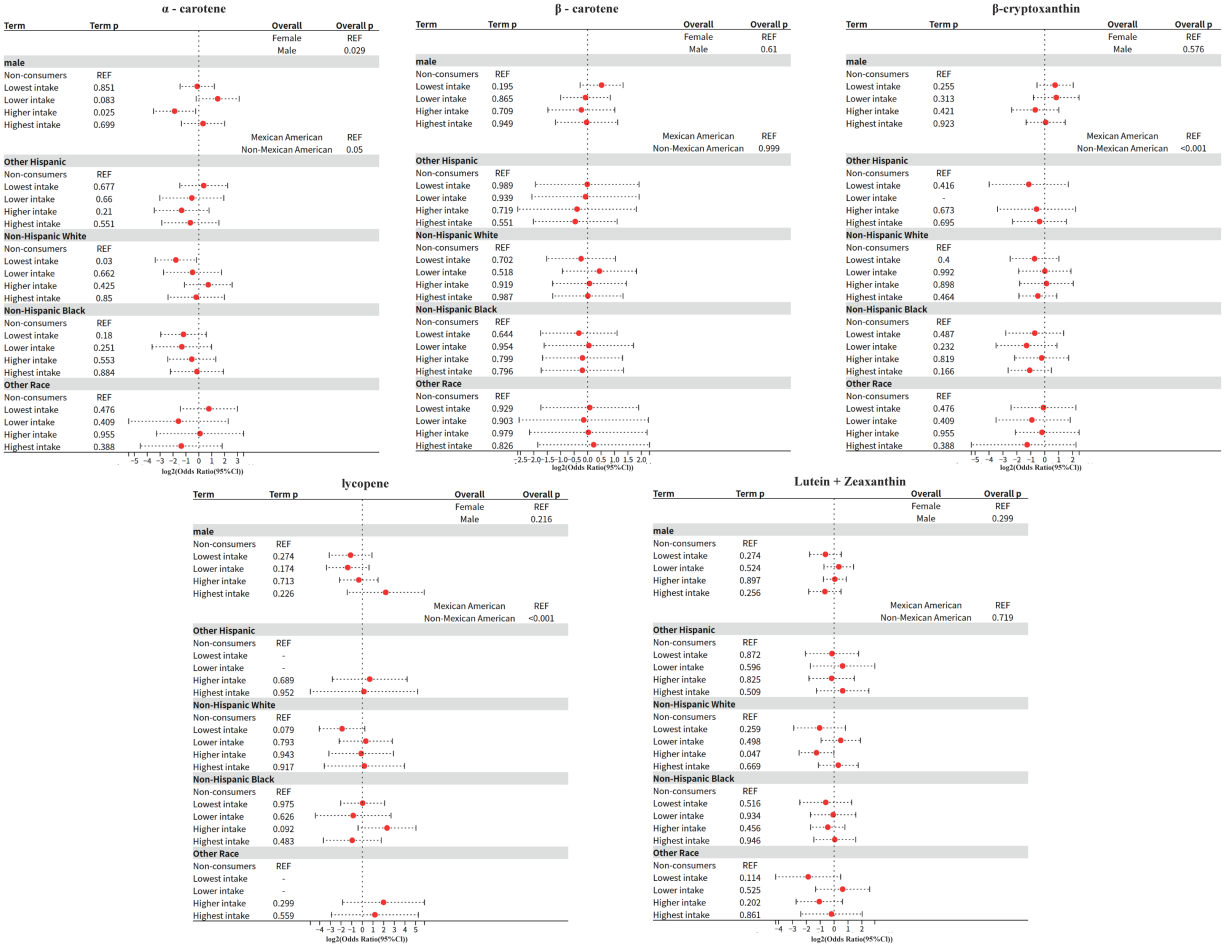
Model 3: Interaction effect.

**Figure 5:**
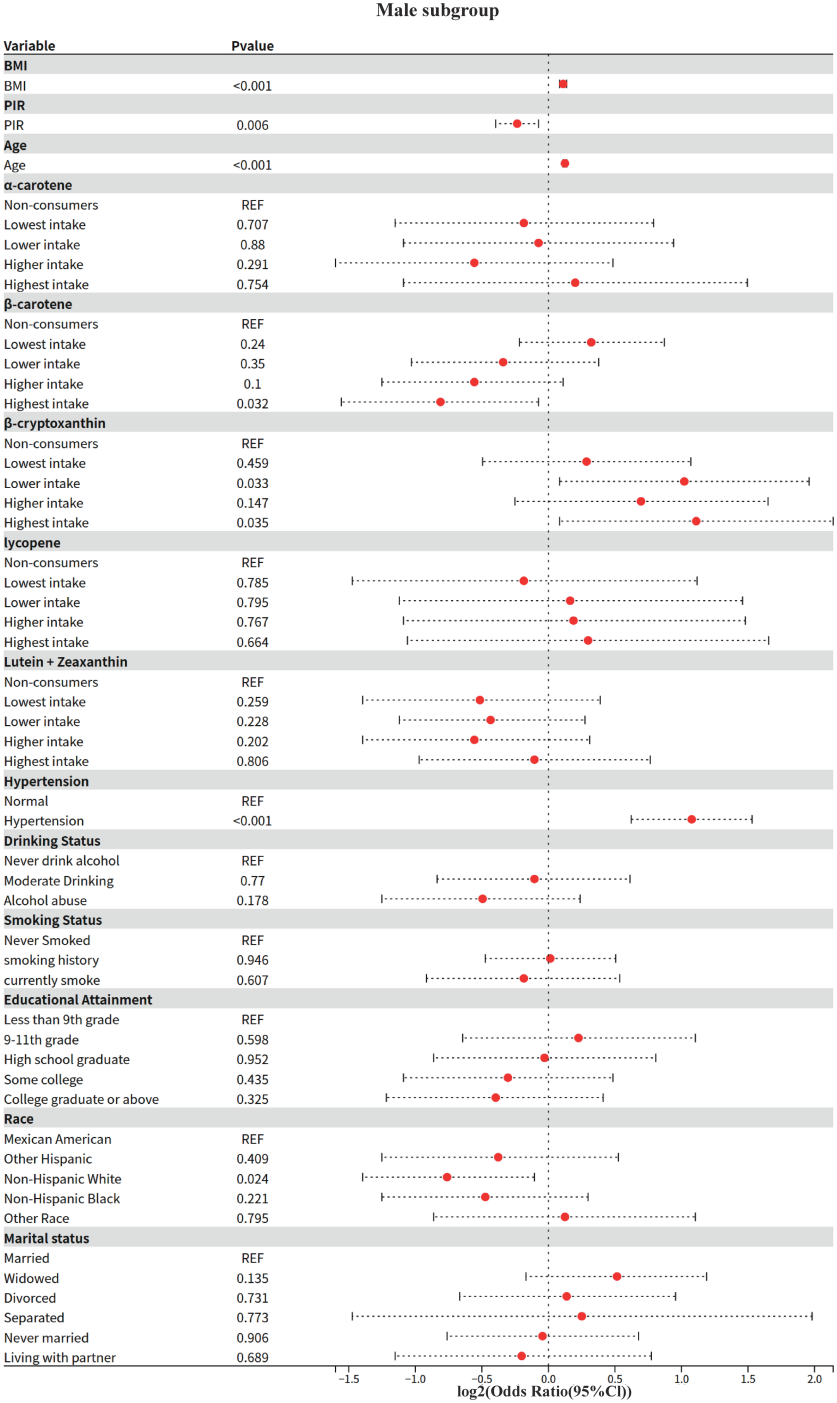
Model 4: Male subgroup.

**Figure 6:**
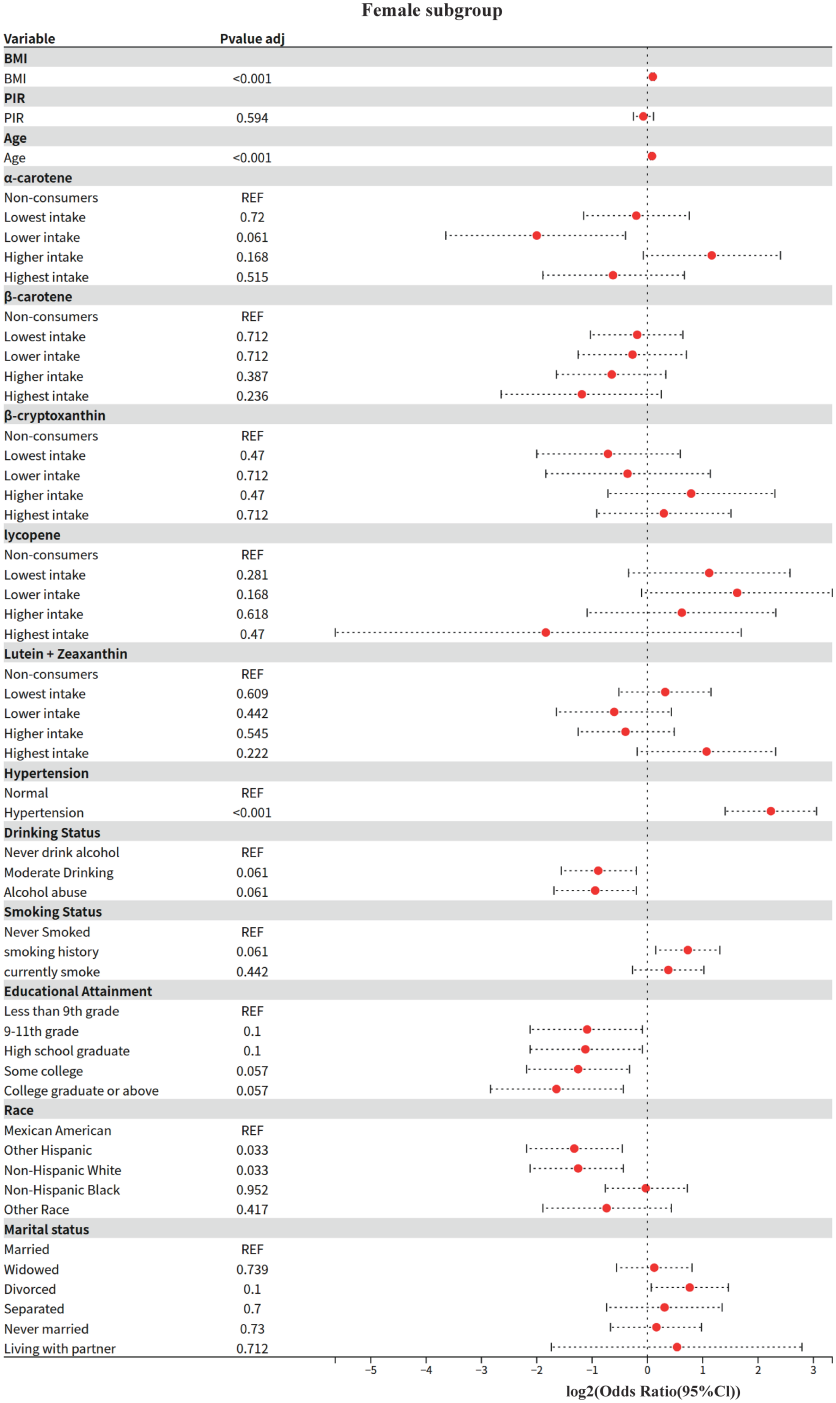
Model 4: Female subgroup.

Based on a nationally representative complex sample design, this study elucidated the significant nutrient-specific dose–response heterogeneity, sex-dependent effect modification, and ethnicity-specific risk patterns between dietary carotenoid intake and the risk of diabetic nephropathy (DN) through multivariable-adjusted models. Response heterogeneity, sex-dependent effect modification, and ethnicity-specific risk patterns between dietary carotenoid intake and the risk of diabetic nephropathy (DN). This finding not only expands the body of evidence for the nutritional management of diabetic nephropathy, but also suggests that clinical risk assessment needs to incorporate a nutritional-demographic interaction perspective, which provides an evidence-based basis for the development of precise nutritional intervention strategies based on complex survey designs (Figures 1–6).

In a multifactorial adjusted model, *α*-carotene intake was significantly and nonlinearly associated with the risk of diabetic nephropathy: the low intake group (OR = 0.25) showed a strong protective effect, while the protective effect disappeared in the highest intake group (OR = 0.65, *p* = 0.339). The molecular basis of this “J-type association” may be related to the following mechanisms:

(1) Protective effect at low doses: efficient antioxidant activity of short conjugated chains.

Novikov et al. showed by Raman spectroscopy and DFT calculations that α-carotene has the shortest conjugated chain among carotenoids (9 fully conjugated double bonds + 1 partially conjugated), and its C=C stretching band experimental value (1,521 cm-^1^) is significantly higher than that of β-carotene (1514–1,516 cm-^1^) (23). The short conjugated chain results in a lower degree of *π*-electron delocalization (elevated calculated wave number), which allows it to be preferentially quenched by single-linear state oxygen (^1^O₂) at low doses, which in turn exerts an efficient antioxidant effect.

Loss of effect at high doses: antioxidant capacity saturation and pro-oxidant transformation.

Nitti pointed out that antioxidants may trigger pro-oxidant effects due to electron donor depletion (“antioxidant paradox”) at high concentrations (24). *α*-Carotene’s short conjugated chain limits its maximum free radical scavenging capacity, and when ingested in amounts above a threshold, unmetabolized α-Carotene may generate secondary oxidative products (e.g., 4-hydroxynonenoic acid) via a lipid peroxidation chain reaction, leading to oxidative damage (24) and consequently loss of protective effects.

In contrast, the highest β-cryptoxanthin intake group showed a statistically significant elevated risk in univariate analysis (OR = 1.41), but the strength of the association was significantly weakened and lost statistical significance after multivariate adjustment (OR = 1.23, *p* = 0.622). This result suggests that the initially observed elevated risk may not be a biological effect of β-cryptoxanthin per se, but rather due to other confounding factors (25). This finding has important implications for clinical nutrition practice: when designing nutrition intervention programs, it is important to systematically assess an individual’s overall health status and potential confounders, and to avoid simplistic associations of single-nutrient intake levels with health risks.

The present study found, for the first time, a significant interaction between alpha-carotene and sex (*p* = 0.029), with the protective effect of higher intake in men being only 1/4 of that in women (OR = 0.27 vs. OR = 1.08 in women). This finding echoes previous studies on sex differences in carotenoid bioavailability, where Stuetz et al. (26) found a significant sex-specific distribution pattern of plasma *α*-carotene levels in a population-based study in six European countries.

This sex difference may originate from the regulation of carotenoid metabolizing enzyme activities by sex hormones. Li et al. (27) demonstrated by *in vitro* experiments that carotenoid cleavage enzyme (CCD4) activity directly affects the metabolic pathway of α-carotene. Proteomic analysis by Yamaguchi et al. (28) further revealed that sex differences in plasma apo-proteins may affect the fat-soluble carotenoid transport and metabolism.

In the present study, it was observed that β-carotene showed a significant protective effect only in males (OR = 0.57), a result consistent with sex-specific metabolic regulation mechanisms in animal experiments (29, 30). Males may enhance the conversion of β-carotene to retinol by upregulating BCO1/BCO2 enzyme activity (29, 31), which in turn activates estrogen receptor signaling pathways (e.g., ERα/ERβ) to inhibit adipogenesis and improve lipid metabolism (32). In addition, the metabolic compensatory effect of males on β-carotene (29) may be more significant by increasing the levels of antioxidant substances (e.g., retinoic acid) and thus benefiting from antioxidant therapy. This mechanism echoes the findings in population studies that cardiovascular disease risk in men is strongly associated with oxidative stress (26, 33). Notably, β-cryptoxanthin showed a dose-dependent increased risk in men (next lowest/highest intake OR = 2.03–2.16), a gender difference that suggests that androgens may promote pro-inflammatory metabolic transformation of this nutrient.

This sex-specific protective effect is not an isolated phenomenon in carotenoid research; Koch et al. (34) observed sex differences in the association of *α*-carotene with cognitive function in their Alzheimer’s disease study, and the aging cohort study by Weber et al. (35) showed a significant sex-specific trajectory of age-related carotenoid level decline. Together, these findings support the possibility that the sex hormone microenvironment may influence the activity of key metabolic enzymes, such as BCO1, through epigenetic regulation, which in turn leads to gender dimorphism in *α*-carotene biological effects. This mechanistic hypothesis provides an important theoretical basis for the development of gender-differentiated nutritional intervention strategies.

Race interaction analysis revealed that the protective effect of minimum α-carotene intake in Non-Hispanic White was only 30% of that in Mexicans (OR = 0.29), a finding that reveals biological heterogeneity in carotenoid metabolism between races, the mechanism of which may involve multilevel interactions: differences in the composition of the intestinal flora Mexican-origin populations have a relatively high abundance of intestinal flora of the phylum Bacteroidetes. The relative abundance of Bacteroidetes (36), which encodes a β-carotene dioxygenase activity that is significantly stronger than that of Firmicutes, the dominant group in Non-Hispanic White (37), is higher in the intestinal flora of Mexican-origin populations.

In contrast, lycopene showed a trend toward higher risk in the higher intake group among Non-Hispanic Black (OR = 4.99), which is highly consistent with the high prevalence of diabetic nephropathy among African-Americans as characterized by the U. S. renal disease epidemiology (38, 39), suggesting that there may be a synergistic effect of genetic predisposition (e.g., APOL1 gene variant) and nutrients. These findings provide an important rationale for precision nutritional interventions: e.g., targeting Afro-descendant populations need to be alert to the risk of high-dose lycopene intake.

Although the interaction model showed a specific protective effect of *α*-carotene in men (OR = 0.48, 95% CI: 0.31–0.75) and Non-Hispanic White (OR = 0.54, 95% CI: 0.33–0.89), the results are doubly challenged: first, the β-cryptoxanthin (OR = 0.001) versus lycopene (OR = 4.59) extreme estimates suggest data segregation (e.g., zero events for some subgroups) or model convergence failures that need to be optimized by Firth correction or Bayesian *a priori* constraints; and second, the wide confidence intervals for Hispanic subgroups (e.g., lycopene CI: 0.28–57.22) expose the inadequacy of the depth of NHANES’ sampling of ethnic minorities, which echoes Paulose-Ram’s pointing out of diversity cohort design flaws (40).

Some of the results of this study differed from the existing literature, such as lutein + zeaxanthin did not show a protective effect, which may be related to the fact that the endpoint event in this study was clinically diagnosed diabetic nephropathy (rather than an early biomarker). Meanwhile, the “protective effect” of alcohol consumption (OR = 0.79, *p* = 0.02 for current drinkers) contradicts conventional wisdom and may reflect the following biases: (1) survival bias: heavy drinkers were not included in the cross-sectional study due to premature death (41); (2) measurement error: the NHANES drinking questionnaire was unable to differentiate between types of red wine (polyphenol protection) and spirits (hepatotoxicity) (42); and (3) Residual confounding: people of higher socioeconomic status are more likely to follow the “red wine in moderation is healthy” dietary pattern. This phenomenon is consistent with that reported by Nielsen et al. (43), but requires precise quantification of exposure by urinary metabolomic markers such as ethylglucosinolate.

Analyses of gender subgroups need to be wary of confounding social factors-higher meat intake in men may synergistically enhance fat-soluble vitamin absorption, whereas women’s pattern of greater reliance on plant-derived diets may impair their bioavailability.

The cross-sectional design of this study limits causal inference, and future prospective cohort validation is warranted. In addition, dietary intake assessment relying on the 24-h retrospective method may be subject to measurement error, but bias has been maximally corrected by weighted analysis. Despite the BH correction, the 216 hypothesis tests for the carotenoid-subgroups may still yield 4–5 false-positive results (expected false discovery rate of 5%), which will need to be repeated to validate the critical significance signals (e.g., alpha-carotene in women *p* = 0.061) by external cohorts. It is worth emphasizing that most of the interactions found in this study existed in small-sample subgroups (e.g., other Hispanic sub-low intake groups), and subsequent studies will need to increase sample size to improve statistical efficacy.

### 4.1 Clinical and policy implications

The findings suggest that clinicians need to be aware when developing nutritional programs for diabetic nephropathy that (1) there may be an optimal dosage window for *α*-carotene intake, (2) male patients are more likely to benefit from β-carotene, and (3) high lycopene diets should be recommended with caution for African Americans. At the public health level, it is recommended that a three-dimensional nutrient-gender-race assessment system be incorporated into diabetes education programs to promote the development of guidelines for individualized nutritional interventions.

## Data Availability

Publicly available datasets were analyzed in this study. This data can be found here: https://wwwn.cdc.gov/nchs/nhanes/Default.aspx.

## Footnotes

^1^ https://www.cdc.gov/nchs/nhanes/

## Supplementary material

Supplementary Figure 1 Normality test.

Supplementary Figure 2 Spearman collinearity analysis.
