# Supplementary figures and images for: The relationship between carotenoids and diabetic nephropathy: insights from NHANES
Source: Front Nutr. 2025 Jul 24;12:1584692. doi: 10.3389/fnut.2025.1584692 (PMC12330217; doi:10.3389/fnut.2025.1584692)

A.Normal distribution

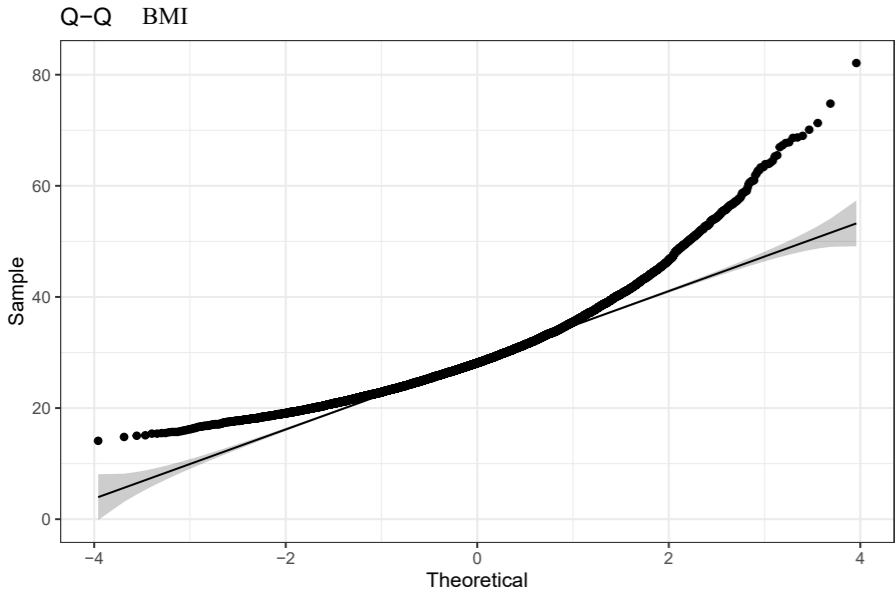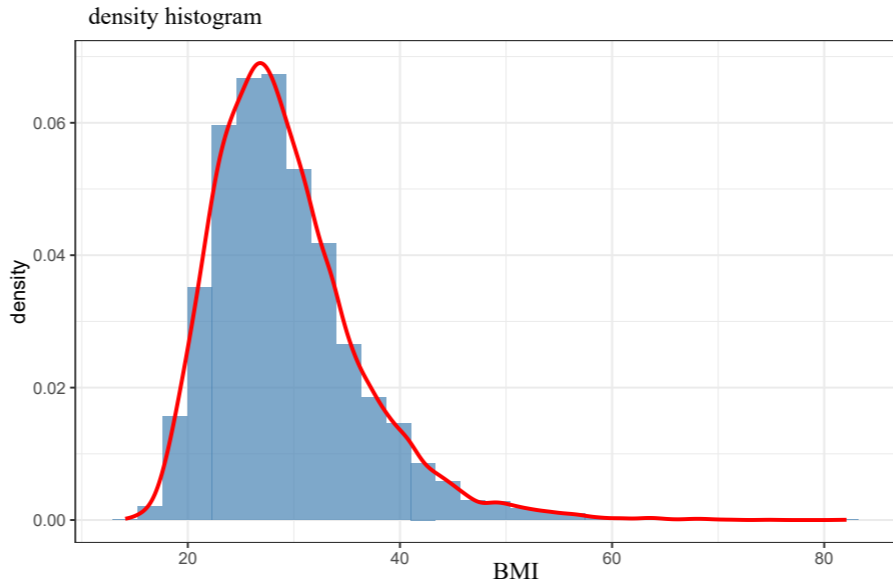

B.Non-normal distribution

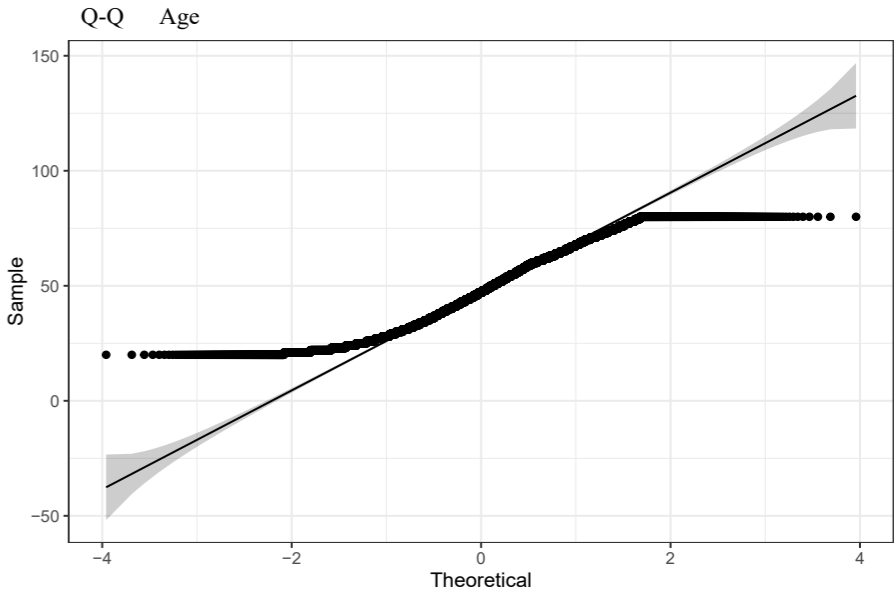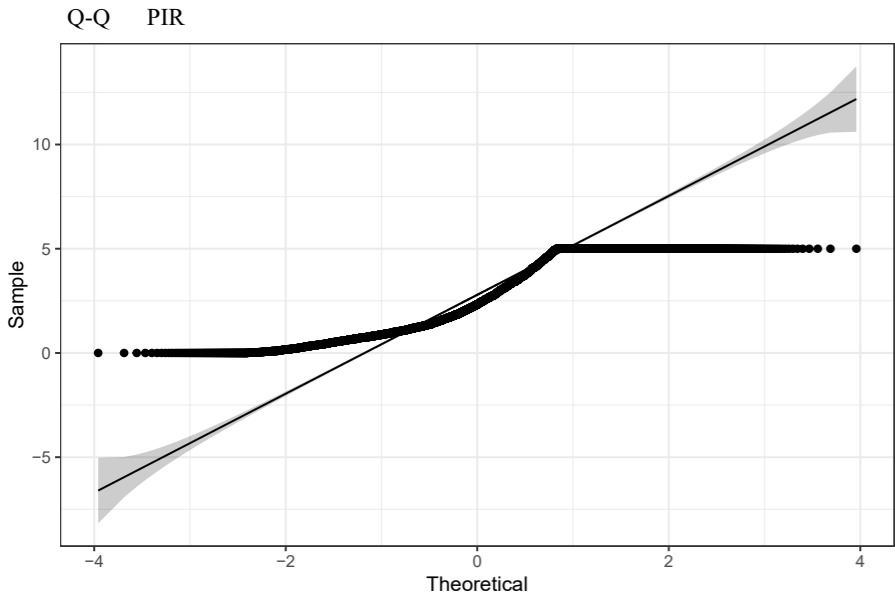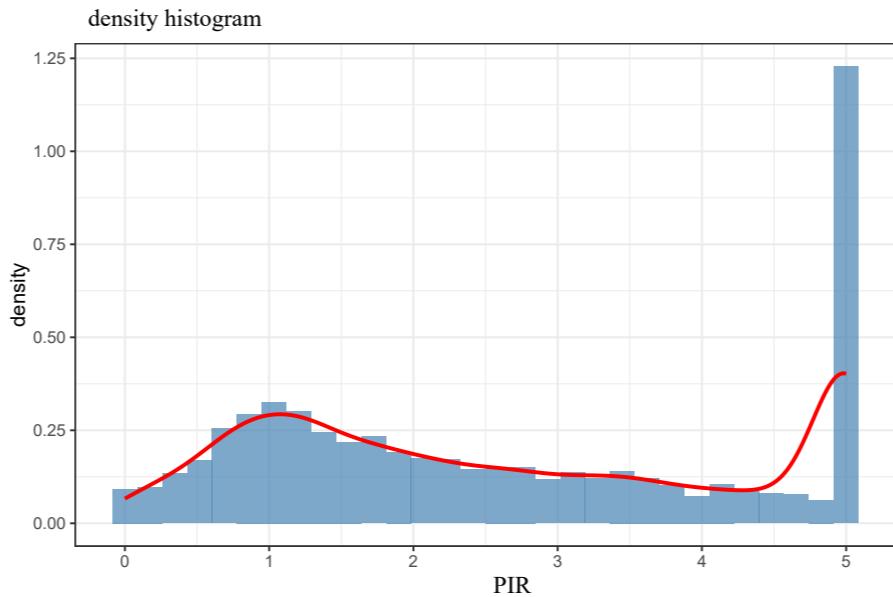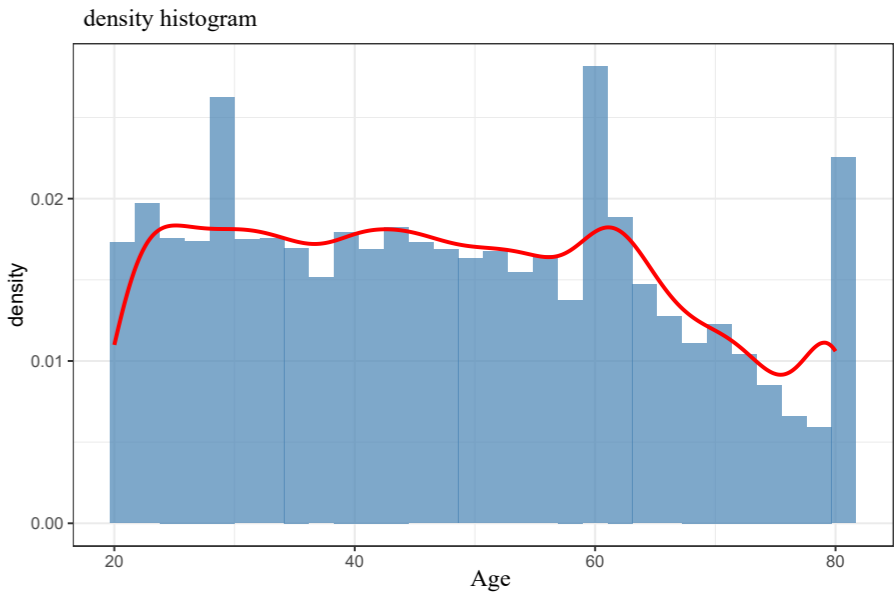

Supplement: Supplementary Figure 1 — Normality test. [file Image_1.pdf]

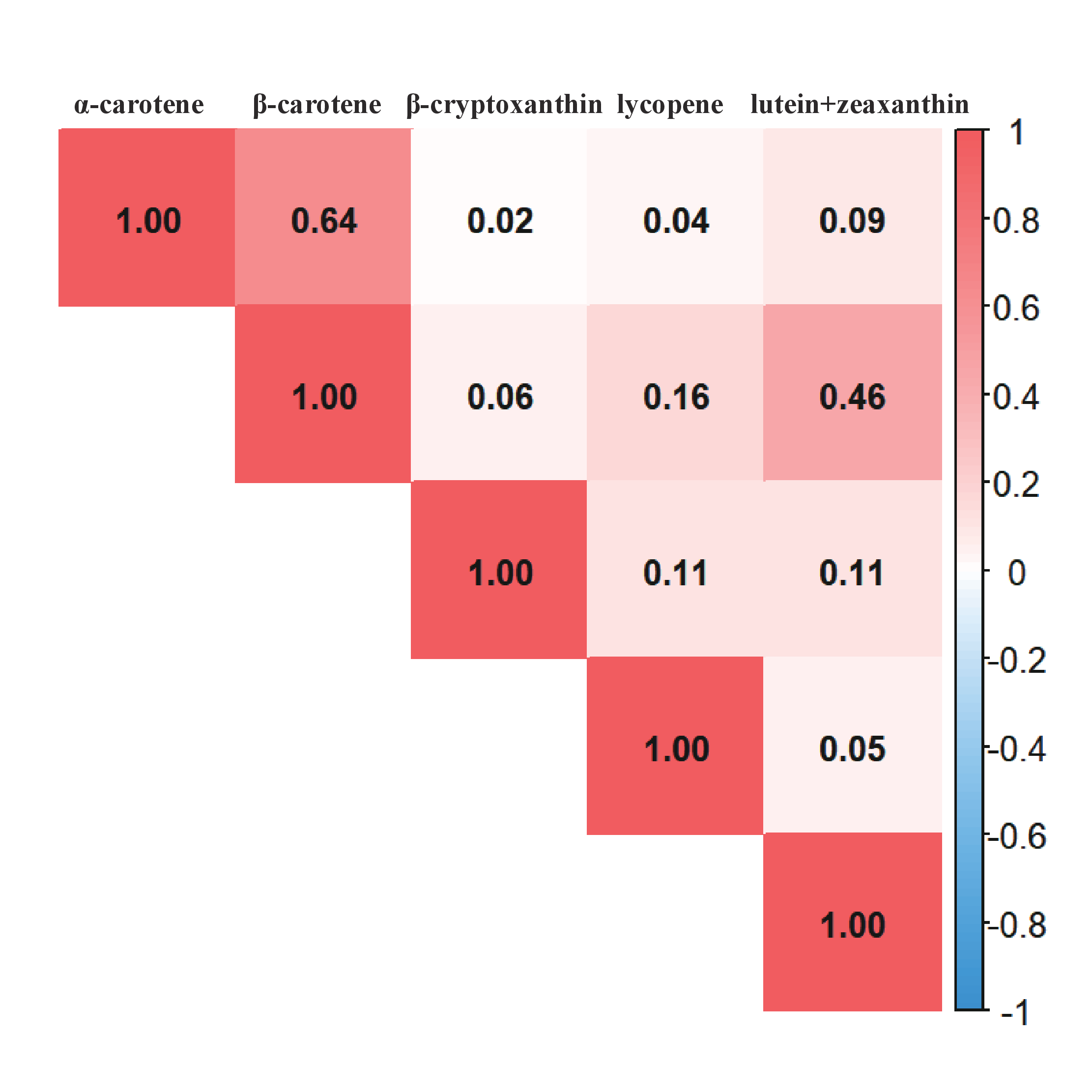

Supplement: Supplementary Figure 2 — Spearman collinearity analysis. [file Image_2.tiff]
